# Methoxy-modified kaolinite as a novel carrier for high-capacity loading and controlled-release of the herbicide amitrole

**DOI:** 10.1038/srep08870

**Published:** 2015-03-09

**Authors:** Daoyong Tan, Peng Yuan, Faïza Annabi-Bergaya, Dong Liu, Hongping He

**Affiliations:** 1 CAS Key Laboratory of Mineralogy and Metallogeny, Guangzhou Institute of Geochemistry, Chinese Academy of Sciences, Guangzhou 510640, China; 2 Key Laboratory of Solid Waste Treatment and the Resource Recycle (SWUST, Ministry of Education), Mianyang 621010, China; 3 Centre de Recherche sur la Matière Divisée, CNRS-Université d’Orléans, Orléans 45071, France; 4 Guangdong Provincial Key Laboratory of Mineral Physics and Materials, Guangzhou 510640, China

## Abstract

Methoxy-modified kaolinite was used as a novel carrier for loading and release of the herbicide 3-amino-1,2,4-triazole, known as amitrole (abbreviated here as AMT). The methoxy modification made the interlayer space of the kaolinite available for AMT intercalation. The AMT loading content in methoxy-modified kaolinite reached up to 20.8 mass% (twice the loading content by unmodified kaolinite). About 48% of this amount is located in the interlayer space. The release profiles of the AMT fit with the modified Korsmeyer-Peppas model. Due to the diffusional restriction of the intercalated AMT by the lamellar structure of the kaolinite and the strong electrostatic attraction between the intercalated AMT and the kaolinite, a slow release of AMT from the methoxy-modified kaolinite was achieved. These results show that the methoxy-modification is a facile method to make the interlayer space of kaolinite available for hosting other guest molecules. The methoxy-modified kaolinite is a promising candidate for high-capacity loading and controlled-release of other molecules such as drugs, agrochemicals, and biochemicals.

Pesticides, mainly insecticides, herbicides, and fungicides, are used worldwide to increase global agricultural productivity, to reduce insect-borne endemic diseases, and to protect plantations^1^. However, the excessive use of pesticide has created serious health problems^2^,^3^ and environmental contaminations^4^. This leads to a great interest in developing controlled-release formulations of pesticides^5^ because such formulations are able to provide safer conditions of use and minimize the potential environmental threat by simultaneously reducing the amount of pesticide used and increasing its efficiency.

Amitrole (3-amino-1,2,4-triazole; AMT) is a non-selective polar herbicide with a wide spectrum of activity against annual and perennial broadleaf and grass-type weeds, acting *via* inhibition of carotenoid biosynthesis^6^. It is of low toxicity to mammals. Owing to its high efficacy in weed control, it is widely used on fallow land prior to sowing, along roadsides and railways, and on wasteland. Because of its water solubility (280 g/L, 25°C) and its low volatility^7^, AMT is readily dissolved in soil solution and is a potential pollution source for the ground water and surface water through leaching, and then contaminate food through plants, fruits, and water media^6^. The development of controlled-release formulations for highly soluble herbicides like AMT is therefore a big requirement, however, some challenges still exist, e.g., high-capacity loading of herbicides is difficult to achieve in agrochemical industry.

Kaolinite is a 1:1 clay mineral consisting of AlO_2_(OH)_4_ octahedral sheet and SiO_4_ tetrahedral sheet. The adjacent sheets are connected together by apical oxygen to form a layer with ideal chemical formulae Al_2_Si_2_O_5_(OH)_4_. The kaolinite layers are linked by hydrogen bonding. Conventionally, kaolinite is rarely used as a carrier for organic compounds such as herbicides despite its much lower cost than most commercialized carriers such as synthetic polymers, because of its low cation exchange capacity (CEC less than 10 mmol/100 g)^8^ and its low specific surface area (SSA between 10 and 20 m^2^/g)^9^. When kaolinite was used as carrier, the guest molecules (e.g. salicylic acid, ibuprofen) could not be intercalated in the interlayer space, but were only adsorbed on the external surfaces of the kaolinite^10^,^11^, resulting in a very low loading content of the guest molecules. For instance, the loading content of the salicylic acid on kaolinite was as low as 5.5 mass%^10^.

At our knowledge, the methoxy-modified kaolinite was used for the first time as carrier for the loading and release of the intercalated AMT herbicide. Attention was focused on the mechanism of the methoxy-modification on the AMT loading and on its release from kaolinite.

## Results

### XRD results

**Figure 1:**
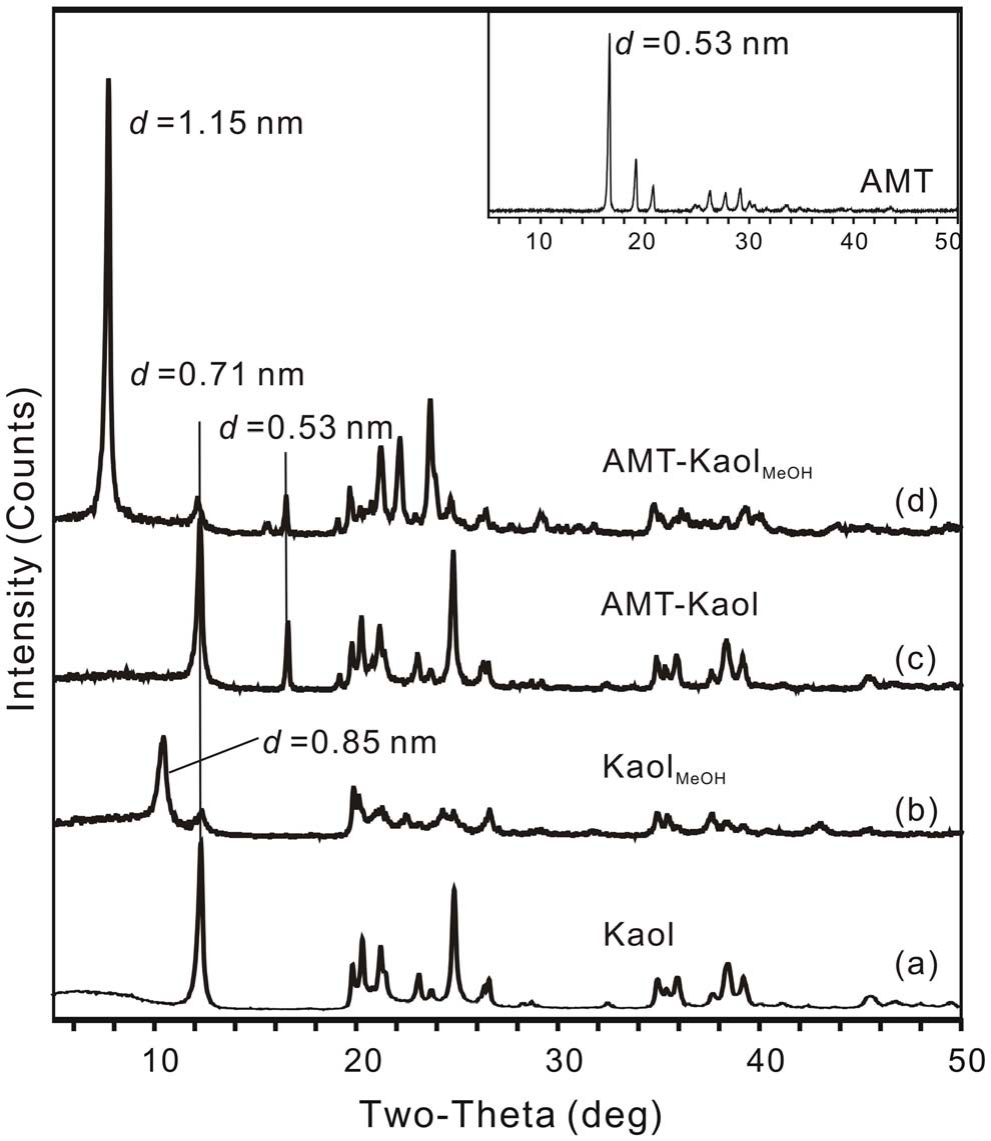
XRD patterns of the kaolinite samples.

The raw kaolinite (Kaol) shows a typical diffraction pattern with a characteristic *d*_001_ value of 0.71 nm (Fig. 1a). Grafting of methoxy groups in the interlayer space of kaolinite^12^ shows a *d*_001_ value in methoxy-modified kaolinite (Kaol_MeOH_) of 0.85 nm (Fig. 1b). The remaining reflection at 0.71 nm in Kaol_MeOH_ is due to a residual unintercalated kaolinite. In the case of AMT-loaded unmodified kaolinite (AMT-Kaol), the *d*_001_ value of kaolinite remains unchanged (Fig. 1c), indicating that AMT cannot intercalate into the interlayer space of the unmodified kaolinite. In the case of the AMT-loaded modified kaolinite (AMT-Kaol_MeOH_) (Fig. 1d), the *d*_001_ value increased to 1.15 nm. Compared to the *d*_001_ value of 0.85 nm observed for Kaol_MeOH_, this increase of 0.30 nm suggests the intercalation of AMT in the interlayer space. The intercalation of AMT is a fast process, a maximum intercalation was achieved in approximately 10 minutes (see details from the Supporting Information, Fig. S1). Although the intercalated AMT increased the interlayer distance of kaolinite, the methoxy modification and AMT loading did not appear to affect the porosity of kaolinite (Table S1, Fig. S2) because the increase of the interlayer distance of kaolinite was not sufficiently large to accommodate the adsorption of N_2_ molecules (0.364 nm) in the interlayer space. However, due to the intercalation of AMT in the interlayer space of the methoxy-modified kaolinite, the AMT loading content in AMT-Kaol_MeOH_, calculated from the percentage of N, reached up to 20.8 mass%, which is twice the loading content of AMT-Kaol (10.3 mass%).

In the XRD patterns of the AMT-loaded kaolinite (Fig. 1c & d), the reflection that corresponds to a *d* value of 0.53 nm is assigned to the (020) crystallographic plane of AMT crystallite (JCPDS card No. 44-1816). This reflection is used to calculate the mean diameter (*D*) of AMT crystallites from the Scherrer equation. The calculated *D* values of the AMT crystallites in AMT-Kaol and AMT-Kaol_MeOH_ are 31.4 and 33.4 nm, respectively. Sliwinska-Bartkowiak *et al*.^13^ have conducted a crystallization study of nitrobenzene in a confined space of porous glasses and MCM-41. Both the experiments and the molecular simulations suggested that the guest was amorphous in the presence of pores with diameters smaller than 15σ, where σ is the diameter of the guest molecule. Based on this characterization, the intercalated AMT was assumed to be amorphous because the interlayer space of the methoxy-modified kaolinite was not large enough for AMT crystallization. This inference also means that the reflection at 0.53 nm represents the AMT crystallites loaded on the external surface of kaolinite.

### IR results

**Figure 2:**
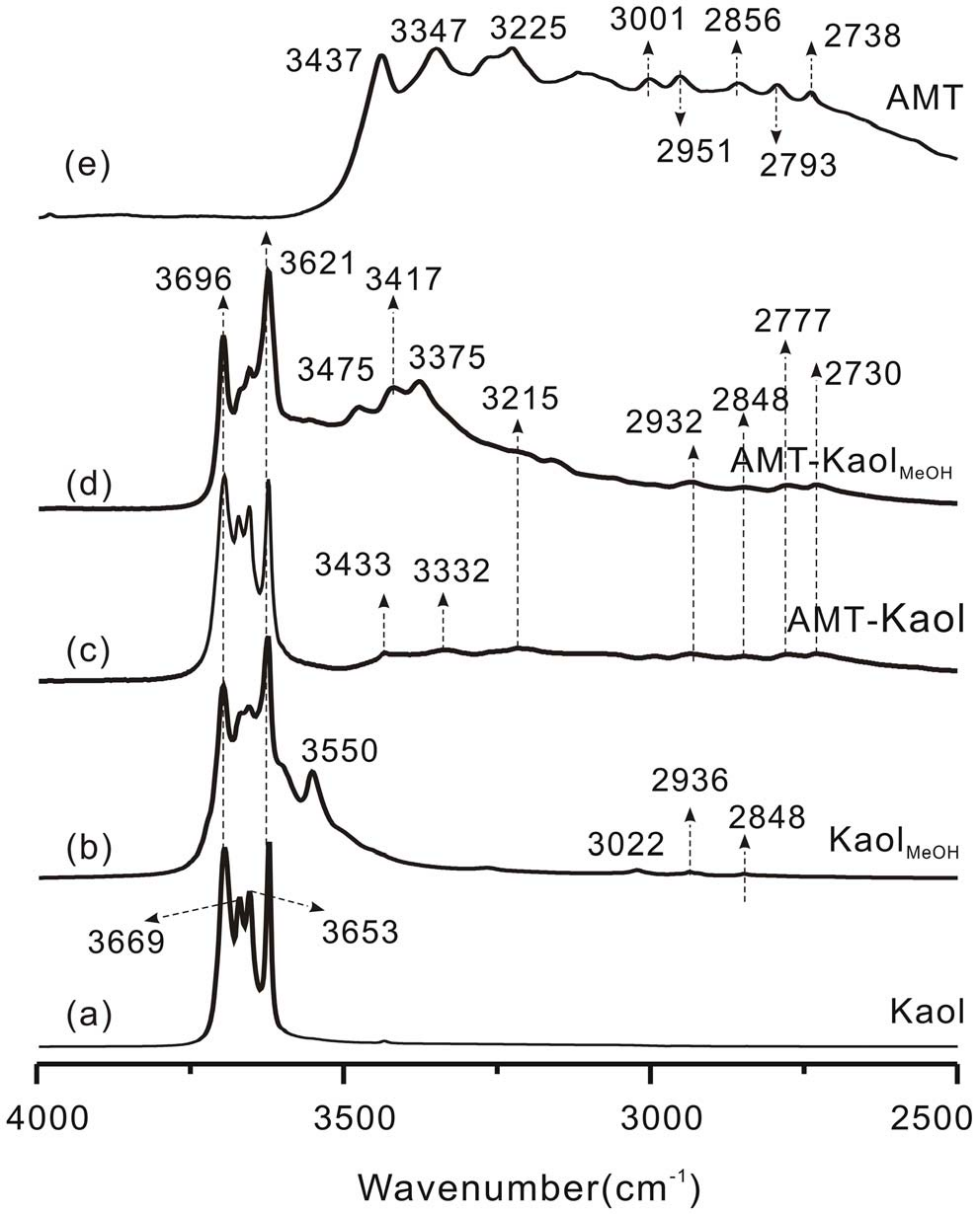
DRIFT spectra of kaolinite and the AMT-loaded kaolinite samples.

The DRIFT spectrum of Kaol (Fig. 2a) exhibits three stretching vibrations of the inner-surface hydroxyl (AlOH) groups at 3696, 3669, and 3653 cm^−1^, as well as the stretching vibration of the inner hydroxyl groups at 3621 cm^−1^. In the DRIFT spectrum of Kaol_MeOH_ (Fig. 2b), the intensities of the three inner-surface hydroxyl groups were weakened. During the methoxy modification one methanol molecule condensed with one AlOH group to form Al-O-C bonding and one H_2_O molecule^12^, leading to a consumption of inner-surface AlOH groups. Also, C-H stretching vibrations at 3022, 2936 cm^−1^, and 2848 cm^−1^ emerged confirming the grafting of methoxy groups on kaolinite. The 3550 cm^−1^ band is characteristic of hydrated kaolinite^14^ indicating that hydrogen bonds between AlOH groups and interlayer water molecules were present.

In the DRIFT spectrum of AMT-Kaol (Fig. 2c), typical vibrations of AMT, including 3433, 3332, 3215, 2932, 2848, 2777, and 2730 cm^−1^, are observed, suggesting the loading of AMT on kaolinite. However, the frequency of these vibrations was lower than those in pure AMT (Fig. 2e). This redshift of the AMT vibrations is caused by the formation of hydrogen bonding between AMT and kaolinite. In the DRIFT spectrum of AMT-Kaol_MeOH_ (Fig. 2d), the intensities of the three inner-surface hydroxyl groups were substantially decreased, indicating the AlOH groups were further consumed during the intercalation of AMT. The new vibration bands at 3475 and 3375 cm^−1^, occurring in the DRIFT spectrum of AMT-Kaol_MeOH_ are assigned to the stretching vibration of the protonated amino group^15^,^16^, which formed *via* the proton transfer from the inner-surface AlOH groups of kaolinite to the amino groups of AMT. Therefore, the interaction between the intercalated AMT and the methoxy-modified kaolinite is an electrostatic attraction, which is stronger than the hydrogen bonding in AMT-Kaol. The vibration band at 3417 cm^−1^ is ascribed to the N-H stretching vibration (Fig. 2d). By comparison with the N-H stretching vibration at 3437 cm^−1^ in pure AMT (Fig. 2e), the redshift of 20 cm^−1^ of the N-H stretching vibration at 3417 cm^−1^ in AMT-Kaol_MeOH_ suggests that the intercalated AMT also formed hydrogen bonding with kaolinite. This redshift is more intense than that observed in AMT-Kaol (redshifted by 4 cm^−1^, from 3437 cm^−1^ to 3433 cm^−1^) (Fig. 2c). This observed higher redshift should be additionally attributed to the stronger electrostatic attraction between the intercalated AMT and the methoxy-modified kaolinite.

### TG and DSC results

**Figure 3:**
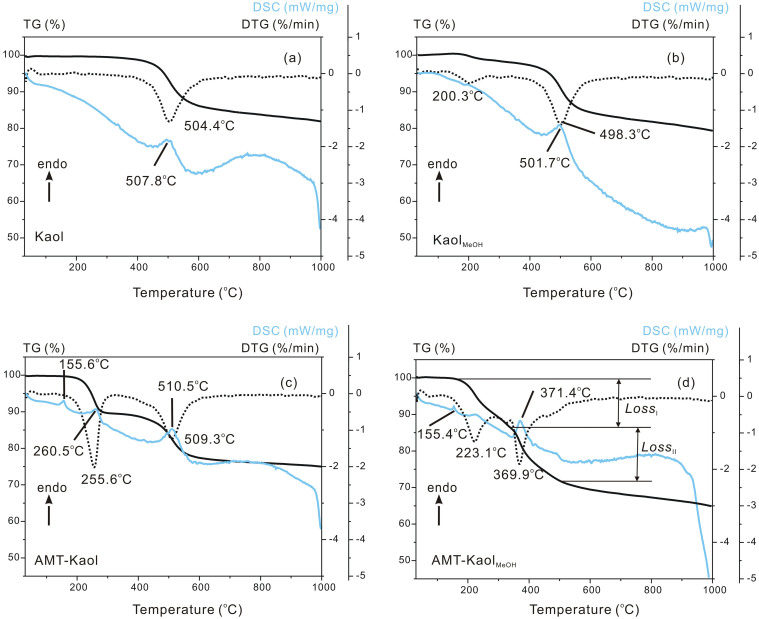
TG, DTG, and DSC curves of (a) Kaol, (b) Kaol_MeOH_, (c) AMT-Kaol, and (d) AMT-Kaol_MeOH_.

In the TG curve of Kaol (Fig. 3a), the major mass loss at approximately 400 to 600°C (endothermic peak at 507.8°C in the DSC curve) is attributed to dehydroxylation of the structural AlOH groups. Two mass losses are observed in the TG curve of Kaol_MeOH_ (Fig. 3b). Because the methoxy groups are stable up to 350°C, the first slow mass loss at approximately 100 to 300°C is attributed to the dehydration of the physically adsorbed water and the interlayer water generated by the condensation between methanol molecules and AlOH groups of kaolinite^12^,^17^. The second substantial mass loss at approximately 300 to 600°C represents the decomposition of the grafted methoxy groups and the dehydroxylation of kaolinite.

In the TG curve of AMT-Kaol (Fig. 3c), the mass loss from 180 to 330°C (endothermic peak at 260.5°C in the DSC curve) is attributed to the decomposition of AMT. The mass loss from 400 to 600°C (endothermic peak at 510.5°C) is associated with the dehydroxylation of kaolinite and the decomposition of the residual AMT (Fig. S3). The small endothermic peak at 155.6°C in the DSC curve is attributed to the melting of AMT crystals.

In the TG curve of AMT-Kaol_MeOH_ (Fig. 3d), two steps of mass loss are clearly resolved. The first mass loss (*Loss*_I_) from 180 to 340°C is attributed to the dehydration of the interlayer water and the decomposition of the non-intercalated AMT adsorbed on the external surface of kaolinite. The second mass loss (*Loss*_II_) from 340 to 500°C (endothermic peak at 371.4°C in the DSC curve) probably resulted from three simultaneous thermal phenomena: i) the loss of the grafted methoxy groups, ii) the dehydroxylation of kaolinite, and iii) the decomposition of the intercalated AMT. The decomposition temperature of the intercalated AMT (340 ~ 500°C) is higher than the decomposition temperature of the non-intercalated AMT (180 ~ 340°C), indicating that AMT is more thermally stable when it is intercalated.

In the case of AMT-Kaol_MeOH_, the respective mass losses of the non-intercalated AMT (from 180 to 340°C) and of the intercalated AMT (from 340 to 500°C) did not overlap. Also, the decomposition of the residual non-intercalated AMT between 500 and 750°C (Fig. S3) did not overlap with the mass loss of the intercalated AMT. Therefore, the relative proportions of the intercalated and non-intercalated AMT in AMT-Kaol_MeOH_ can be estimated by determining the ratio between their corresponding mass losses in the TG curve. Following this method, the calculated amounts in AMT-Kaol_MeOH_ were respectively 9.9 mass% for the intercalated AMT and 10.9 mass% of the non-intercalated AMT. These results mean there was approximately 47.6% AMT located in the interlayer space and 52.4% AMT adsorbed on the external surface of the kaolinite.

### Controlled release results

**Figure 4:**
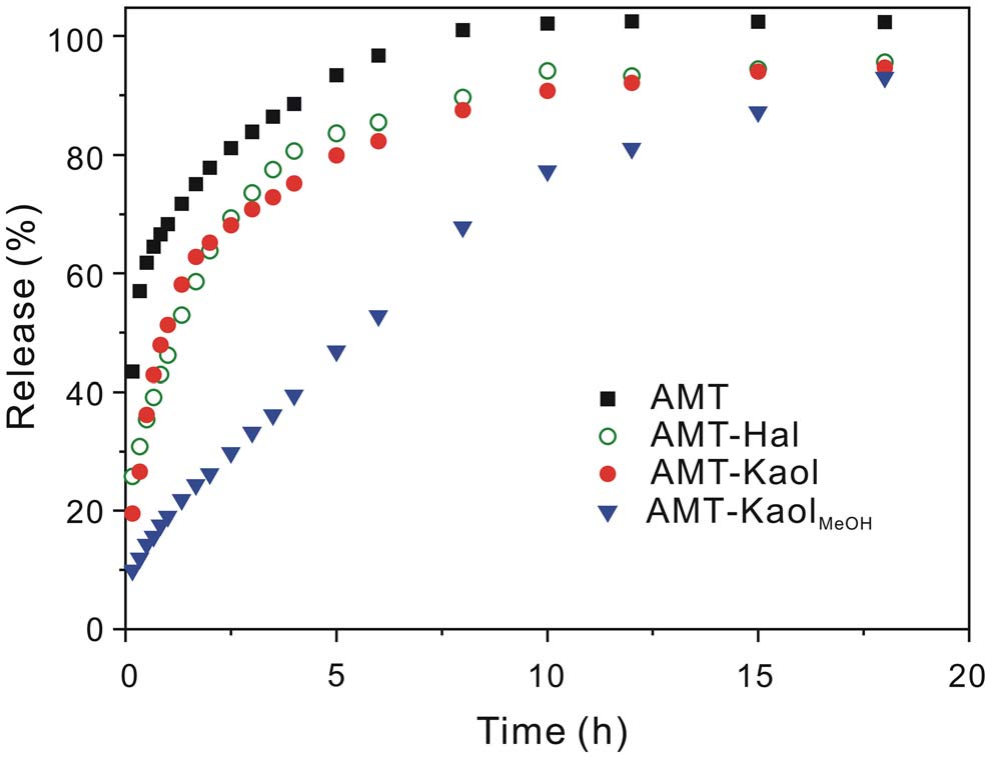
release profiles of AMT from clay minerals.

The release profiles of AMT are presented in Fig. 4. Because of the high water solubility of AMT, pure AMT exhibited a rapid release with a burst effect, with a large amount of AMT (>50%) released over a very short period of time (< 30 min). Compared to the release of pure AMT, the release of AMT from AMT-Kaol was only slightly retarded. This slightly retarded release of AMT is ascribed to the hydrogen bonding between AMT and kaolinite. However, the release of AMT from AMT-Kaol_MeOH_ was intensively retarded, and the burst effect of AMT was significantly attenuated. These results suggest that the methoxy modification of kaolinite had a positive effect by slowing the release of AMT from the kaolinite.

The release kinetics of AMT from kaolinite samples was evaluated by fitting the release profiles to the modified Korsmeyer-Peppas model^18^,^19^:
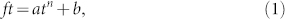
where *a* is a constant that incorporates the structural and geometric characteristics of the drug dosage form; *n* is the release exponent characteristic of the release mechanism; and *b* represents the burst effect in the release. And the first 60% of each release profile was used for the simulation. In this model, Fickian diffusion through a slab is indicated by a diffusional exponent (*n*) of 0.50, while an anomalous transport (non-Fickian) mechanism is indicated by an *n* value of 0.50 ~ 1.0. The parameters of this equation for each AMT release profile and the corresponding coefficients of determinations (*R^2^*) are listed in Table 1. The high values of *R^2^* (> 0.989) suggest that the release profiles of AMT were well fitted using the modified Korsmeyer-Peppas model. The *a* value for AMT-Kaol_MeOH_ is 0.11, much smaller than the *a* value for AMT-Kaol (0.68), indicating a much slower release of AMT from AMT-Kaol_MeOH_ than that from AMT-Kaol. The *n* values for AMT-Kaol is 0.36, lower than 0.50, indicating that some other release mechanism occured^20^. However, the *n* value for AMT-Kaol_MeOH_ is 0.79, indicating a non-Fickian diffusion mechanism. All these results suggest that the methoxy modification of the kaolinite influence the release mechanism of AMT.

## Discussion

The intercalation of AMT in the methoxy-modified kaolinite leads to an interlayer distance of 0.30 nm which was slightly smaller than the height of AMT with molecular dimensions approximately 0.75 nm × 0.62 nm× 0.37 nm (length × width × height)^7^. As one third of the inner-surface hydroxyl groups of kaolinite were grafted by methoxy groups^12^, the AMT molecules were probably intercalated in a horizontal monolayer arrangement in which the plane of the five-membered heterocyclic of AMT was parallel to the kaolinite layers, and part of the AMT molecule was keyed into the plane of the grafted methoxy groups.

The intercalated amorphous AMT exhibited higher thermal stability than the non-intercalated crystalline AMT because the intercalated AMT i) formed strong electrostatic attraction with kaolinite, and ii) could be protected from the thermal decomposition by the lamellar structure of the kaolinite. This finding is in agreement with the higher thermal stability of other intercalated organic guests that have been found to decompose at higher temperatures in the interlayer space of clay minerals, such as cationic surfactant in montmorillonite^21^ and organosilane in silane-grafted kaolinite^22^.

The methoxy-modification allowed the intercalation of AMT in the interlayer space of kaolinite, and significantly promoted the loading capacity till 20.8 mass%. This value is much higher than the AMT loading that has been achieved on montmorillonite (1.5 mass%)^23^. The reason for the low loading of AMT on montmorillonite is that AMT has a p*K*a value of 4.1 and is not positively charged in a wide range of pH. So, on montmorillonite it is difficult to load AMT through cation exchange but AMT was primarily adsorbed as neutral molecules and exhibited a low level of loading^23^. Although some other swelling clay minerals and organoclays are commonly used as carriers for the loading of pesticides, one drawback of these carriers is that they normally exhibit low loading capacities for pesticides. For example, organoclays have been found to exhibit loadings of only approximately 5.5 mass% for hexazinone^24^, 7.7 mass% for 2,4-dichlorophenoxyacetic acid^25^, and 12.5 mass% for bentazone^26^.

Moreover of the high-capacity loading of AMT, AMT-Kaol_MeOH_ also exhibited a controlled-release of AMT, which was achieved *via* the two previously indicated routes: the diffusional restriction of the intercalated AMT by the lamellar structure of kaolinite and the strong electrostatic attraction of the intercalated AMT to kaolinite. Generally, tubular halloysite, chemically and structurally similar to kaolinite, is used as carrier for loading and release of various guests^27^,^28^,^29^,^30^,^31^,^32^,^33^,^34^. The guest, mainly encapsulated into the lumen of halloysite, presented a controlled-release behavior because its diffusion was retarded by the halloysite tubular structure. However, in this study, the release of AMT from halloysite (AMT-Hal) was much faster than the release of AMT from the methoxy-modified kaolinite (Fig. 4). This result suggests that the diffusion-restriction of the lamellar structure of kaolinite is even more excellent than the tubular structure of halloysite, which endows the methoxy-modified kaolinite with a controlled-release property. Additionally, it can be anticipated that this controlled-release property could be readily further promoted using a combination of some other techniques, such as commonly used tabletting^35^ and coating techniques^27^,^29^.

Previous results and discussion show that the promotion of the loading and controlled-release of AMT by methoxy-modification on kaolinite succeeded to act with the following mechanism. The methoxy-modification introduced a plane of methoxy groups in the interlayer space of kaolinite weakening the original hydrogen bonding between the adjacent layers. This interlayer space became available for further AMT intercalation. Moreover, the lamellar kaolinite structure could significantly restrict the diffusion of the intercalated AMT, leading to its slow release from the modified kaolinite.

The methoxy-modified kaolinite is a potentially promising carrier for the long-term controlled release of certain specific guest molecules, enabling promising prospects in future applications. For example, because kaolinite is widely used in paint, the intercalation of some anticorrosives (benzotriazole or 8-hydroxyquinoline) or antimicrobials into the interlayer space of the kaolinite could possibly endow the paint with long-acting anticorrosion or antimicrobic functions *via* the slow release of these anticorrosives or antimicrobials agents. Other chemicals such as drugs, agrochemicals, and biochemicals of similar functional groups and molecular dimensions to AMT could also achieve high loading and controlled release in methoxy-modified kaolinite.

In summary, the methoxy modification of kaolinite could develop an additional interlayer space for loading of pesticides, drugs, and some other bioactive chemicals. The methoxy-modified kaolinite loaded with these chemicals also allowed their controlled release.

## Methods

### Material

Amitrole (AMT) was purchased from Meryer (99%). All other reagents were of analytical grade. A high-purity kaolinite sample, obtained from Guangdong Province, China, was used as received without further purification and labeled as Kaol.

### Methoxy-modification of kaolinite

Dimethyl sulfoxide (DMSO) was first intercalated into the interlayer space of Kaol as previously reported^22^. Then, 5.0 g of the DMSO-intercalated kaolinite sample was added to 100 mL of methanol (MeOH) and stirred for 7 days. The solids in the mixture were separated *via* centrifugation and then stored in a wet state for further use. The methoxy-modified kaolinite was labeled as Kaol_MeOH_.

### Loading of AMT on kaolinite by soaking methods

A sample of 2.0 g of AMT was dissolved in 20 mL of MeOH, and approximately 1.0 g of each used clay mineral, before and after methoxy modification, was added under constant stirring for 24 h at room temperature. The solid part of the dispersion was separated *via* centrifugation and dried overnight at 80°C. The AMT-loaded kaolinite samples were identified by adding the prefix “AMT-” to the starting materials; for example, AMT-Kaol_MeOH_ refers to the kaolinite that was modified with MeOH and then loaded with AMT.

### AMT-release test

The AMT-release tests were conducted using an RCZ-8M dissolution tester (Tianjin TDTF Technology Co. Ltd., China) following a paddle method. Approximately 0.5 g of AMT-loaded clay mineral sample was sealed in a dialysis bag (molecular mass cutoff: 3500 Da), and then soaked in 500 mL of distilled water at room temperature with a rotation speed of 100 rpm. At suitable intervals, 5 mL of the dissolution medium was withdrawn, and an equivalent volume of fresh medium was added. The AMT content was determined at 239 nm using a PerkinElmer LAMBDA 850 UV/Vis spectrophotometer. Each dissolution test was performed in triplicate. After the achievement of 100% release of AMT, the clay mineral samples were collected for thermal analysis to calculate the amount of the intercalated AMT.

### Characterization

The X-ray diffraction (XRD) patterns were obtained using a Bruker D8 Advance diffractometer with a Ni filter and Cu Kα radiation (λ = 0.154 nm) generated at 40 kV and 40 mA. Diffuse reflectance infrared Fourier-transform (DRIFT) spectra were obtained using a Bruker Vertex 70 Fourier-transform infrared spectrometer at room temperature. The CHN elemental analyses were performed using an Elementar Vario EL III Universal CHNOS Elemental Analyzer. Thermogravimetry (TG) and differential scanning calorimetry (DSC) were performed with a Netzsch STA 409PC instrument. Transmission electron microscopy (TEM) observations were recorded using a 200 kV JEOL JEM-2100 high-resolution transmission electron microscope. Nitrogen adsorption-desorption isotherms were measured using a Micromeritics ASAP 2020 instrument at liquid nitrogen temperature.

## Author Contributions

D.Y.T. and P.Y. conceived and designed the experiments. D.Y.T. and D.L. analyzed the data. D.Y.T. and P.Y. wrote the manuscript. F.A.B. and H.P.H. are involved in the related discussion and F.A.B. helps to improve the quality of the manuscript.

## Supplementary Material

Supplementary Information SI

## Figures and Tables

**Table 1:** Parameters of modified Korsmeyer-Peppasmodel

	*ft* = a*t*^n^ *+ b*			
Samples	*a*	*n*	*b*	*R^2^*
AMT-Kaol	0.68	0.36	−0.17	0.9892
AMT-Kaol_MeOH_	0.11	0.79	0.079	0.9986

## References

[b1] Ecobichon D. J. Pesticide use in developing countries. Toxicology 160, 27–33 (2001). 11246121 10.1016/s0300-483x(00)00452-2

[b2] Rothlein J. *et al.* Organophosphate pesticide exposure and neurobehavioral performance in agricultural and nonagricultural Hispanic workers. Environ. Health. Persp. 114, 691–696 (2006). 10.1289/ehp.8182 PMC1459921 16675422

[b3] Nasterlack M. Pesticides and childhood cancer: An update. Int. J. Hyg. Envir. Heal. 210, 645–657 (2007). 10.1016/j.ijheh.2007.03.001 17434797

[b4] Atreya K., Sitaula B. K., Johnsen F. H. & Bajracharya R. M. Continuing issues in the limitations of pesticide use in developing countries. J. Agr. Environ. Ethic. 24, 49–62 (2011).

[b5] Nennemann A. *et al.* Clay-based formulations of metolachlor with reduced leaching. Appl. Clay Sci. 18, 265–275 (2001).

[b6] Oesterreich T., Klaus U., Volk M., Neidhart B. & Spiteller M. Environmental fate of amitrole: Influence of dissolved organic matter. Chemosphere 38, 379–392 (1999). 10901662 10.1016/s0045-6535(98)00185-4

[b7] Fontecha-Camara M. A., Lopez-Ramon M. V., Pastrana-Martinez L. M. & Moreno-Castilla C. Kinetics of diuron and amitrole adsorption from aqueous solution on activated carbons. J. Hazard. Mater. 156, 472–477 (2008). 18241982 10.1016/j.jhazmat.2007.12.043

[b8] Ma C. & Eggleton R. A. Cation exchange capacity of kaolinite. Clay. Clay Miner. 47, 174–180 (1999).

[b9] Castellano M. *et al.* Bulk and surface properties of commercial kaolins. Appl. Clay Sci. 48, 446–454 (2010).

[b10] Bonina F. P. *et al.* Adsorption of salicylic acid on bentonite and kaolin and release experiments. Appl. Clay Sci. 36, 77–85 (2007).

[b11] Mallick S. *et al.* Formation of physically stable amorphous phase of ibuprofen by solid state milling with kaolin. Eur. J. Pharm. Biopharm. 68, 346–351 (2008). 17669640 10.1016/j.ejpb.2007.06.003

[b12] Tunney J. J. & Detellier C. Chemically modified kaolinite. Grafting of methoxy groups on the interlamellar aluminol surface of kaolinite. J. Mater. Chem. 6, 1679–1685 (1996).

[b13] Sliwinska-Bartkowiak M. *et al.* Melting/freezing behavior of a fluid confined in porous glasses and MCM-41: Dielectric spectroscopy and molecular simulation. J. Chem. Phys. 114, 950–962 (2001).

[b14] Tunney J. & Detellier C. Preparation and characterization of an 8.4-angstrom hydrate of kaolinite. Clay. Clay Miner. 42, 473–476 (1994).

[b15] Heacock R. A. & Marion L. The infrared spectra of secondary amines and their salts. Can. J. Chem. 34, 1782–1795 (1956).

[b16] Muresan-Pop M. *et al.* Spectroscopic and physical-chemical characterization of ambazone-glutamate salt. Spectrosc-Biomed App. 26, 115–128 (2011).

[b17] Komori Y. *et al.* Modification of the interlayer surface of kaolinite with methoxy groups. Langmuir 16, 5506–5508 (2000).

[b18] Kim H. & Fassihi R. Application of binary polymer system in drug release rate modulation 2. Influence of formulation variables and hydrodynamic conditions on release kinetics. J. Pharm. Sci. 86, 323–328 (1997). 9050800 10.1021/js960307p

[b19] Costa P. & Sousa Lobo J. M. Modeling and comparison of dissolution profiles. Eur. J. Pharm. Sci. 13, 123–133 (2001). 11297896 10.1016/s0928-0987(01)00095-1

[b20] Sopena F., Cabrera A., Maqueda C. & Morillo E. Controlled release of the herbicide norflurazon into water from ethylcellulose formulations. J. Agr. Food Chem. 53, 3540–3547 (2005). 15853399 10.1021/jf048007d

[b21] He H. P. *et al.* Thermal characterization of surfactant-modified montmorillonites. Clay. Clay Miner. 53, 287–293 (2005).

[b22] Yang S. *et al.* Effect of reaction temperature on grafting of γ-aminopropyl triethoxysilane (APTES) onto kaolinite. Appl. Clay. Sci. 62–63, 8–14 (2012).

[b23] Morillo E., Perezrodriguez J. L. & Maqueda C. Mechanisms of interaction between montmorillonite and 3-aminotriazole. Clay Miner. 26, 269–279 (1991).

[b24] Celis R., Hermosin M. C., Carrizosa M. J. & Cornejo J. Inorganic and organic clays as carriers for controlled release of the herbicide hexazinone. J. Agr. Food Chem. 50, 2324–2330 (2002). 11929292 10.1021/jf011360o

[b25] Hermosin M. C. *et al.* Bioavailability of the herbicide 2,4-D formulated with organoclays. Soil Biol. Biochem. 38, 2117–2124 (2006).

[b26] Carrizosa M. J., Calderon M. J., Hermosin M. C. & Cornejo J. Organosmectites as sorbent and carrier of the herbicide bentazone. Sci. Total Environ. 247, 285–293 (2000). 10803556 10.1016/s0048-9697(99)00498-2

[b27] Kelly H. M., Deasy P. B., Ziaka E. & Claffey N. Formulation and preliminary in vivo dog studies of a novel drug delivery system for the treatment of periodontitis. Int. J. Pharm. 274, 167–183 (2004). 15072793 10.1016/j.ijpharm.2004.01.019

[b28] Veerabadran N. G., Price R. R. & Lvov Y. M. Clay nanotubes for encapsulation and sustained release of drugs. Nano 2, 115–120 (2007).

[b29] Veerabadran N. G., Mongayt D., Torchilin V., Price R. R. & Lvov Y. M. Organized shells on clay nanotubes for controlled release of macromolecules. Macromol. Rapid Commun. 30, 99–103 (2009). 21706582 10.1002/marc.200800510

[b30] Abdullayev E. & Lvov Y. Halloysite clay nanotubes for controlled release of protective agents. J. Nanosci. Nanotechno. 11, 10007–10026 (2011). 10.1166/jnn.2011.5724 22413340

[b31] Vergaro V., Lvov Y. M. & Leporatti S. Halloysite clay nanotubes for resveratrol delivery to cancer cells. Macromol. Biosci. 12, 1265–1271 (2012). 22887783 10.1002/mabi.201200121

[b32] Yuan P., Southon P. D., Liu Z. W. & Kepert C. J. Organosilane functionalization of halloysite nanotubes for enhanced loading and controlled release. Nanotechnology 23, 375705 (2012). 22922808 10.1088/0957-4484/23/37/375705

[b33] Tan D. *et al.* Natural halloysite nanotubes as mesoporous carriers for the loading of ibuprofen. Micropor. Mesopor. Mat. 179, 89–98 (2013).

[b34] Tan D. *et al.* Loading and in vitro release of ibuprofen in tubular halloysite. Appl. Clay Sci. 96, 50–55 (2014).

[b35] Sharma C., Rana A. C., Bala R., & Seth N. An overview of industrial process validation of tablets. J. Drug Deliv. Ther. 3, 175–183 (2013).

