# Supplementary material for: Methoxy-modified kaolinite as a novel carrier for high-capacity loading and controlled-release of the herbicide amitrole
Source: Sci Rep. 2015 Mar 9;5:8870. doi: 10.1038/srep08870 (PMC4352846; doi:10.1038/srep08870)
Supplement: Supplementary Information — SI [file srep08870-s1.pdf]

Methoxy-modified kaolinite as a novel carrier for high-capacity loading and controlled-release of the herbicide amitrole

Daoyong TAN[1,2], Peng YUAN[1*,4], Faïza ANNABI-BERGAYA[3], Dong LIU[1,4], Hongping HE[1,4]

[1] CAS Key Laboratory of Mineralogy and Metallogeny, Guangzhou Institute of Geochemistry, Chinese Academy of Sciences, Guangzhou 510640, China

[2] Key Laboratory of Solid Waste Treatment and the Resource Recycle (SWUST, Ministry of Education), Mianyang 621010, China

[3] Centre de Recherche sur la Matière Divisée, CNRS-Université d'Orléans, Orléans 45071, France

[4] Guangdong Provincial Key Laboratory of Mineral Physics and Materials, Guangzhou 510640, China

[*] Corresponding authors: Peng YUAN (yuanpeng@gig.ac.cn)

First author: Daoyong TAN (tdyduff@hotmail.com)

Supporting Information

The AMT adsorption kinetics

To study the kinetics of adsorption of AMT on kaolinite, a series of adsorption of AMT was conducted within different adsorption time, ranged from 10 minutes to 24 hours. The obtained products were identified by adding postfix "-X" to AMT-Kaol$_{MeOH}$, for example, AMT-Kaol$_{MeOH}$-10min refers to that the adsorption time was 10 minutes.

The intercalation of AMT is a fast process, and a maximum intercalation was achieved in approximately 10 minutes because the (001) reflection at 0.85 nm for the methoxy-modified kaolinite was not observed in the XRD pattern of AMT-Kaol$_{MeOH}$-10min (Fig. S1a). It is hard to investigate the kinetics of adsorption of AMT by fitting classic kinetic models due to the lack of effective data, which was caused by the fast intercalation and adsorption of AMT on kaolinite. Instead, we studied the adsorption kinetics by comparison with the AMT loading content between kaolinite samples with different adsorption time. The AMT loading content of AMT-Kaol$_{MeOH}$-10min was 18.0 mass%. The non-intercalated AMT was 8.1 mass%, which was obtained by subtracting the amount of intercalated AMT (9.9 mass%, see details in the following part) from the total AMT loading content. This value was lower than that in AMT-Kaol$_{MeOH}$-24h (10.9 mass%), indicating an unsaturated adsorption of AMT onto the surface of kaolinite. The complete adsorption of AMT onto the surface of kaolinite could be achieved within 30 minutes because the AMT loading content of AMT-Kaol$_{MeOH}$-30min was 20.8 mass%, which was equal to that of AMT-Kaol$_{MeOH}$-24h. For the samples of different adsorption time (from 30 minutes to 24 hours), the loading content of AMT was fluctuated within a

narrow range. In this study, the adsorption time was set as 24 hour so as to achieve a steady loading of AMT on kaolinite. In summary, the loading of AMT on kaolinite was fast, including both intercalation of AMT into the interlayer space of kaolinite and adsorption of AMT onto the external surface of kaolinite, and the adsorption time had little effect on the loading of AMT on kaolinite.

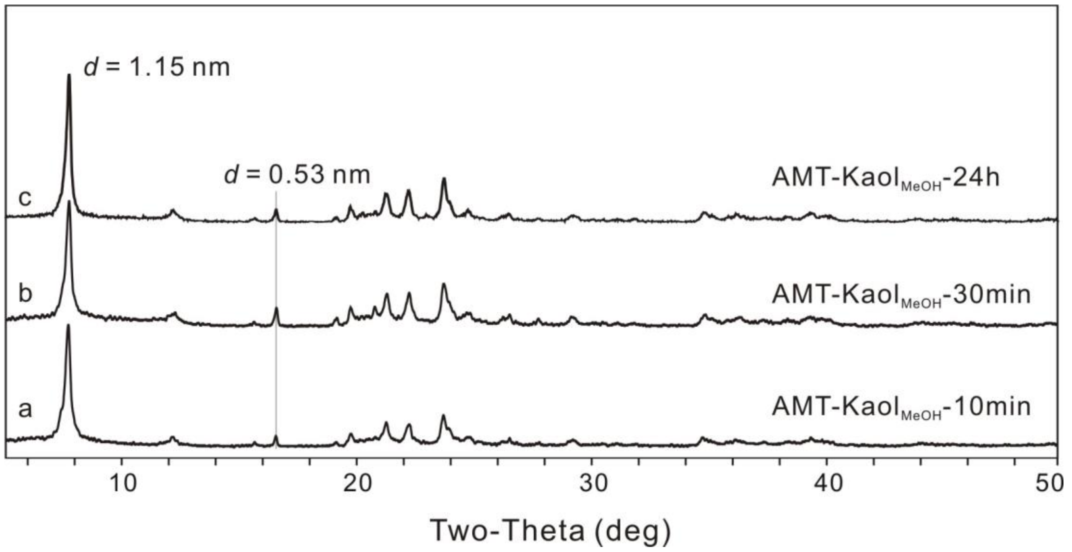

Fig. S1 The XRD patterns of AMT-loaded kaolinite samples.

N_2 adsorption-desorption isotherms analysis

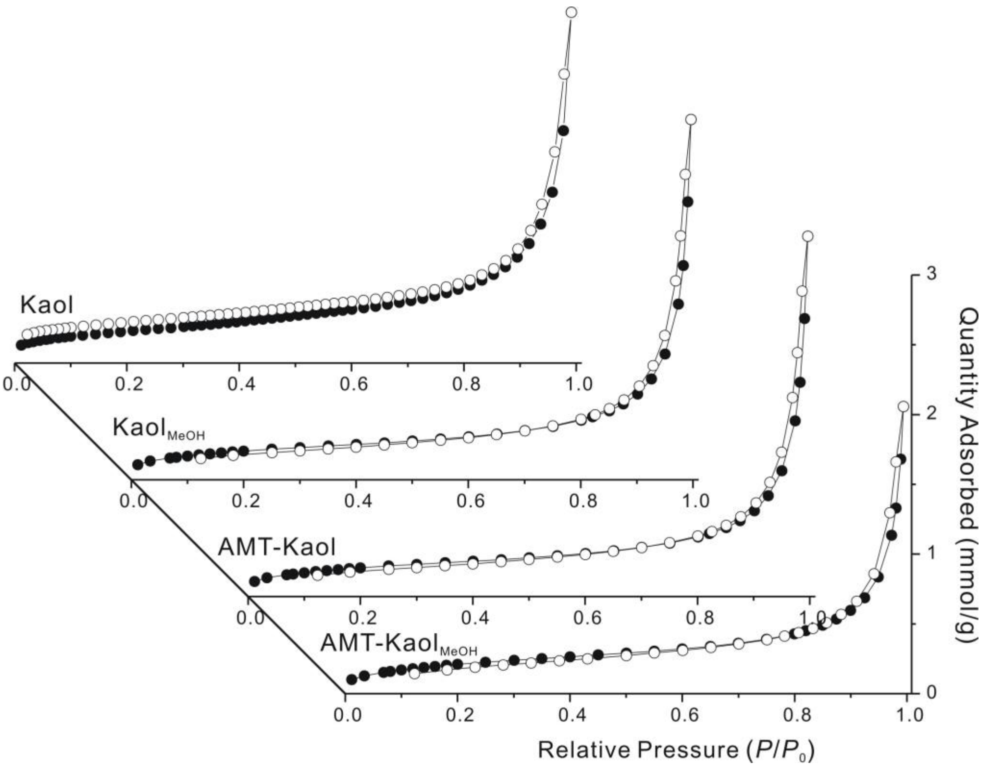

Fig. S2 the N_2 adsorption-desorption isotherms of the kaolinite samples.

According to the IUPAC classification, the N_2 adsorption-desorption isotherms of Kaol resemble type II isotherms with a minor hysteresis loop (Fig. S2), which indicates that the mesopores arising from the stacking of the kaolinite particles are small in scale. The SSA and V_{Pore} values of Kaol are 17.8 m^2/g and 0.08 cm^3/g, respectively. The low level of porosity of Kaol can be attributed to the fact that the main contributions to the SSA and V_{pore} of Kaol are the external surface area and the interparticle pores, respectively. In addition, the interlayer distance of kaolinite is smaller than the molecular diameter of N_2 (0.364 nm), which means that the interlayer surface cannot be detected by N_2 molecules. Although the methoxy modification and the intercalation of AMT into the interlayer space of kaolinite increased the interlayer distance of the

kaolinite, this increase was not sufficiently large to accommodate the adsorption of N_2 molecules in the interlayer space. As a result, the N_2 adsorption-desorption isotherms of the methoxy-modified kaolinite and the AMT-loaded kaolinite (Fig. S2) does not present any visible change in comparison with that of Kaol. This conclusion is also clearly supported by that the SSA and V_{Pore} values of the methoxy-modified kaolinite and the AMT-loaded kaolinite are nearly identical to those of Kaol (Table S1), which means the kaolinite retained its porosity during methoxy modification and AMT loading.

Table S1 SSA and porosity data of kaolinite samples

Samples	SSA (m^2/g)	V_{Pore} (cm^3/g)
Kaol	17.8	0.08
$\mathrm{Kaol_{MeOH}}$	18.1	0.07
AMT-Kaol	16.3	0.09
$\mathrm{AMT\text{-}Kaol_{MeOH}}$	17.1	0.07

Thermal analysis

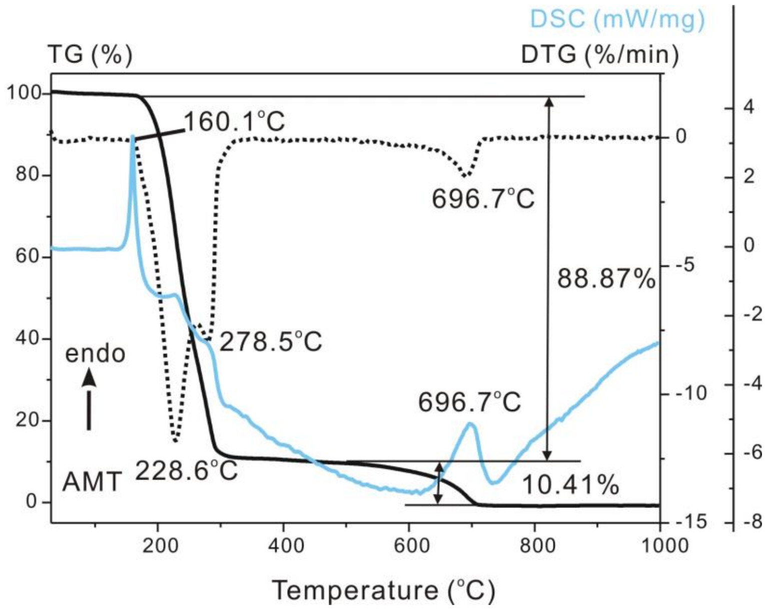

Fig. S3 TG, DTG, and DSC curves of AMT.

The mass loss of the pure AMT occurred in two steps: the first substantial mass loss (88.87%) from 170 to 350°C and the second slight mass loss (10.41%) from 500 to 750°C. The endothermic peak at 160.1°C in the DSC curve is attributed to the melting of AMT crystallites.

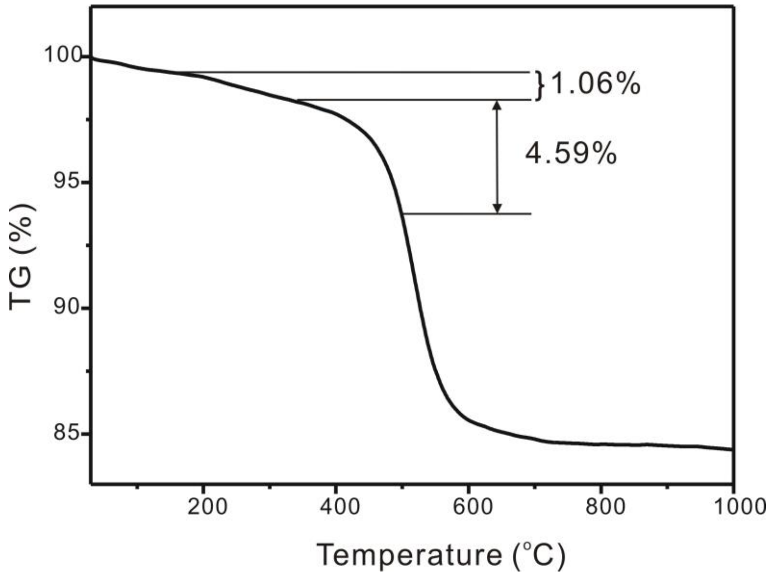

Fig. S4 TG curve of the AMT-released AMT-Kaol$_{MeOH}$.

The amounts of the non-intercalated AMT and the intercalated AMT in AMT-Kaol$_{MeOH}$ were calculated as follows. In the TG curve of AMT-Kaol$_{MeOH}$, the mass loss from 180 to 340°C ($Loss_I$) is 12.78%, including the loss of the interlayer water and the non-intercalated AMT. The mass loss from 340 to 500°C ($Loss_{II}$) is 14.47%, including the loss of the grafted methoxy groups, the AlOH groups of the kaolinite, and the intercalated AMT. In the TG curve of the AMT released AMT-Kaol$_{MeOH}$ (Fig. S4), the mass loss of the interlayer water in the range of 180 to 340°C is 1.06%, and the mass loss of the grafted methoxy groups and the AlOH groups of the kaolinite in the range of 340 to 500°C is 4.59%. The amount of AMT loaded into AMT-Kaol$_{MeOH}$ was 20.8 mass%, which means that the amount of kaolinite in AMT-Kaol$_{MeOH}$ was 79.2 mass%. Thus, the actual mass loss of the interlayer water in $Loss_I$ is 0.84%, and the actual total mass loss of the grafted methoxy groups and the AlOH groups of the kaolinite in $Loss_{II}$ is 3.63%. By subtracting these mass losses from the total mass losses in $Loss_I$ and in $Loss_{II}$, respectively, in the TG curve of AMT-Kaol$_{MeOH}$, the actual mass loss of the non-intercalated AMT and the intercalated AMT can be found to be 11.94% and 10.84%, respectively. Since the relative proportions of the non-intercalated AMT and the intercalated AMT in AMT-Kaol$_{MeOH}$ are identical to the ratio between their corresponding mass losses in the TG curve, therefore, the amount of the non-intercalated AMT was 10.9 mass% in AMT-Kaol$_{MeOH}$, and the amount of the intercalated AMT was 9.9 mass%. It should be noted that the precisely quantitative calculation of the amounts of the intercalated AMT and the non-intercalated AMT is difficult to be achieved, because there

is lack of any clear boundary between the decomposition of the intercalated AMT and the non-intercalated AMT in the TG curve.

TEM analysis

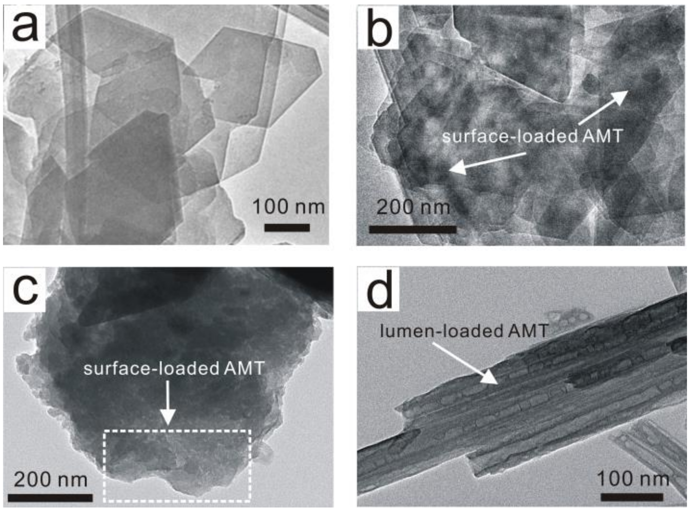

Fig. S5 TEM images of (a) Kaol, (b) AMT-Kaol, (c) AMT-Kaol$_{MeOH}$, and (d) AMT-Hal.

The kaolinite particles in Kaol exhibit a pseudo-hexagonal morphology (Fig. S5a). When simplified as square particles, the kaolinite particles in Kaol have an average size of approximately 500 × 500 ± 200 nm and an average thickness of approximately 400 ± 150 nm, which was determined *via* atomic force microscopy. The loading of AMT on the kaolinite caused an obvious change in the TEM images. The aggregation of AMT on the external surfaces of the kaolinite particles is shown in Fig. S5b & c. The loading of the AMT aggregates is indicated by the different levels of contrast on the TEM images; the darker region (as denoted by the arrows in Fig. S5b) represents the AMT aggregates on the external surface of the kaolinite. In addition, the loading of AMT aggregates causes the edges of the kaolinite particles to become poorly resolved (as denoted by the dots in the box in Fig. S5c). In the TEM image of the AMT-loaded halloysite (Fig. S5d), the lumens of halloysite are blocked by AMT particles, which are interrupted by voids. The discontinuous loading of the AMT into the lumen may have been resulted from the incomplete removal of air from the lumen.
